# Effect of Yoga Exercise on Premenstrual Symptoms among Female Employees in Taiwan

**DOI:** 10.3390/ijerph13070721

**Published:** 2016-07-16

**Authors:** Su-Ying Tsai

**Affiliations:** Department of Health Management, I-Shou University, Kaohsiung, Taiwan No.8, Yida Rd., Yanchao Township, Kaohsiung Country 824, Taiwan; sytsai@isu.edu.tw; Tel.: +886-7-615-1100 (ext. 7414); Fax: +886-7-615-5150

**Keywords:** female employee, premenstrual symptoms, yoga exercise, SF-36

## Abstract

Yoga classes designed for women with premenstrual syndrome are available, but their efficacy is unclear. We investigated the effects of 12 weeks’ yoga exercise (yoga intervention) on premenstrual symptoms in menstruating females in Taiwan. Sixty-four subjects completed the yoga intervention, and before and after the intervention filled out a structured self-report questionnaire about their demographics, personal lifestyle, menstrual status, baseline menstrual pain scores, premenstrual symptoms, and health-related quality of life. Of 64 subjects, 90.6% reported experiencing menstrual pain during menstruation. After the yoga intervention, subjects reported decreased use of analgesics during menstruation (*p* = 0.0290) and decreased moderate or severe effects of menstrual pain on work (*p* = 0.0011). The yoga exercise intervention was associated with the improvement of the scale of physical function (*p* = 0.0340) and bodily pain (*p* = 0.0087) of the SF-36, and significantly decreased abdominal swelling (*p* = 0.0011), breast tenderness (*p* = 0.0348), abdominal cramps (*p* = 0.0016), and cold sweats (*p* = 0.0143). Menstrual pain mitigation after yoga exercise correlated with improvement in six scales of the SF-36 (physical function, bodily pain, general health perception, vitality/energy, social function, mental health). Employers can educate female employees about the benefits of regular exercise such as yoga, which may decrease premenstrual distress and improve female employee health.

## 1. Introduction

Premenstrual syndrome (PMS) is a common disorder in menstruating females. Women with PMS often report cyclical symptoms that are both psychological, such as irritability, and physical, such as headaches and back pain [1]. Up to 80% of females experience at least one premenstrual symptom during their menstrual cycle [2], but are still able to function normally at work and at home. Premenstrual dysphoric disorder (PMDD) is a severe, sometimes disabling variant of premenstrual syndrome (PMS) [3]. Females with PMS report a poorer perceived work-related quality of life in their professional lives [4] and health-related quality of life [5,6], and PMS may result in a depressed mood and greater psychiatric comorbidity [7]. Severe menstrual symptoms can significantly impact the quality of life of the affected women, interfering with school, employment, interpersonal relationships, family, social life [6], and lead to increased healthcare utilization, decreased occupational productivity, and absence from work [8].

Female employees with PMS have decreased job career satisfaction levels and well-being; have more issues balancing family and work commitments, and more stress at work; are less involved in decisions that affect themselves at work; and are less happy about their working conditions [4]. All women, regardless of race, age, or socioeconomic status, have experienced discomfort during their menstrual periods. To our knowledge, when female workers have PMS in the workplace in Taiwan, care personnel in the workplace usually provide heat packs or suggest bed rest at a health center or may provide painkillers. These methods, which merely alleviate female workers’ pain at the time the menstrual cramps occur, are not preventive methods. Hence, more and more workplaces and employers are attempting to determine preventive methods that can ameliorate PMS in female workers during their menstrual periods, and further investigate the correlation between yoga exercise intervention classes in the workplace and indexes of menstrual pain, discomfort during menstrual periods, overall well-being, and quality of life.

PMS is also linked to anxiety and depression [9,10] and is associated with both direct and indirect medical costs due to absenteeism and low productivity in the workplace [11]. The etiology of premenstrual symptoms is uncertain. A wide range of different treatment regimens, including lifestyle changes, complementary and alternative medicine (CAM), and drug therapies are promoted for PMS and PMDD [12]. Exercise is commonly listed as a remedy for PMS. Intervention studies demonstrate that aerobic exercise increases hemoglobin, hematocrit, red cell count, and platelet count, and decreases the levels of prolactin, estradiol, and progesterone; resulting in improvement of fatigue, impaired concentration, confusion, and most other premenstrual symptoms [12,13]. These findings reveal that exercise effectively reduces the symptoms of PMS and can be used as a treatment. Exercise is considered a CAM therapy for improving and maintaining physical and emotional health. A growing body of evidence indicates that yoga benefits physical and mental health by downregulating the hypothalamic-pituitary-adrenal axis and the sympathetic nervous system [14], and yoga has become an increasingly popular form of CAM among people with pain [15,16]. A randomized controlled trial in India demonstrated that Yoga Nidra practice was helpful in patients with hormone imbalances [17]. One study reported that three yoga poses (specifically the cobra, cat, and fish poses) reduced the severity and duration of primary dysmenorrhea [18]. Another study reported that a yoga intervention was associated with a reduction in the severity of dysmenorrhea [19]. Even a simple home-based yoga program available on a DVD was shown to reduce menstrual pain and improve overall health status [20]. Yoga is a mind and body practice with historical origins in ancient Indian philosophy. Many clinicians treating persistent pain hear about the benefits of yoga from patients who frequently go to yoga centers. Yoga classes specifically designed for women with PMS have increased; however, few intervention-based studies have focused on this issue among female workers.

Empirical research on the alleviation of menstrual discomfort through yoga-centered health promotion activities remains insufficient, and no research in Taiwan has addressed improvement or changes in female workers’ menstrual discomfort through interventional psychologic and physical yoga activities in the workplace. Thus, the aim of the present study was to investigate the effect of a 12-week yoga exercise program on premenstrual symptoms in menstruating female workers. We postulated that female workers participating in a regular yoga exercise program would have fewer premenstrual symptoms. The results will contribute to our understanding of the current status of a menstrual health-friendly workplace environment for female employees and can be used to establish a model for a healthy lifestyle with a regular yoga exercise to decrease the negative impact of premenstrual symptoms.

## 2. Methods

### 2.1. Research Setting and Subjects

This was a yoga exercise intervention study for premenstrual symptoms conducted from August 2014 through October 2015 among females employed in a large electronics manufacturer in Taiwan. The research setting was Company C, a large electronics manufacturer in Taiwan. The employer and director of health management pay attention to female employees’ premenstrual disorders. Thus, Company C was willing to provide assistance in administering this study. Out of the 10 manufacturing plants owned by Company C, one was selected for this yoga intervention study of premenstrual symptoms.

Inclusion criteria were: female employees aged 20 to 45 years working in the manufacturing plant who were healthy premenopausal women, taking no oral contraceptives, and taking no medication during the last 3 months. According to the information provided by the director of health management, in 2014 the study plant had 401 female employees aged 20 to 45. After excluding those taking oral contraceptives (21 subjects) and analgesic medications during the last 3 months (111 subjects), 269 subjects were eligible for the study. This study invited all eligible employees to participate in the study. The Institutional Review Board of E-Da Hospital (Taiwan) approved the study (EMRP-104-015). Written and signed informed consent was obtained from each participant. A total of 198 female employees volunteered participated in the study beginning, with a response rate of 73.6%. However, only 64 female employees completed the full 12-week yoga exercise program, and 134 employees did not complete the 12-week course or dropped out.

### 2.2. Study Design and Yoga Exercise Intervention

The yoga exercise program was 12 weeks long and featured physically and psychologically interventional yoga activities to determine changes in female workers’ menstrual discomfort. In this study, the yoga instructor was an experienced professionally certified yoga instructor. We selected a female instructor who understands premenstrual symptoms in consideration of female participants who might feel self-conscious during the exercise program. To encourage successful completion of the 12-week intervention program and to facilitate active engagement of the participants in the study, the yoga instructor, the director of health management, and the researchers determined the optimal yoga protocol, frequency of the yoga class, and class location at the beginning of the study.

The yoga teacher personally guided each participant’s yoga exercise activities twice-a-week 50-min sessions after work in the plant’s fitness center. There were four yoga classes a week to choose from, allowing subjects to select the most convenient time to participate in two yoga classes per week. Each 50-min session comprised a 5-min breathing exercise, a 35-min yoga pose practice, and 10-min supine meditation/relaxation. In this study, we adopted Kapalbhati Pranayama breathing exercises of yoga, and five basic yoga poses (cat-cow, child’s pose, downward dog, plank, and cobra) were included in our yoga protocol. To ensure the safety and adaptability of the female employees, it was agreed upon in the planning stage of the study that only a simple and basic yoga protocol would be practiced in the class, as this would be the first yoga exercise intervention for many of the participants in the study.

Yoga exercise practice sessions begin with a prayer or invocation to create an environment that relaxes the mind. Yoga is typically performed slowly, in a relaxed manner, with awareness of one’s body and respiration. To reap the true benefits from yoga practice, one must master breath control, or Pranayama. Kapalbhati Pranayama breathing exercises were adopted in our study and the Kapalbhati Pranayama technique includes automatic inhalation with short and forceful exhalations. The five basic yoga poses practiced in our study included cat-cow, child’s pose, downward dog, plank, and cobra. Cat-cow pose stretches the abdominal muscles, neck and back, and maintains the flexibility of the spine; this pose is especially helpful for people with stiff backs. Child’s pose stretches the lower back and hips, and helps to relieve stress, decrease back discomfort, fatigue, gas, and bloating. The downward dog pose strengthens arms, shoulders, abdominal and quadriceps muscles, and ankles, while stretching the shoulders, hamstrings, calves, and chest. Plank pose strengthens the arms, wrists, and spine. The cobra pose stretches the chest and abdominal muscles and maintains the flexibility of the spine. It also improves poor posture and combats depression, lower back discomfort, and low energy. The class ends with supine meditation/relaxation. During supine meditation, any meditative posture can be assumed with the eyes closed and the whole body relaxed. The savasana pose (dead body posture), is performed by lying down, face up with the arms and legs comfortably apart and palms facing upward, eyes closed, and the whole body consciously relaxed. These final postures help to relax the whole psycho-physiologic system. The same yoga routine was practiced for 12 weeks.

Before starting the yoga exercise intervention programs, we administered a structured self-report questionnaire to the subjects to collect information about the demographics, personal lifestyle, menstrual status, baseline menstrual pain scores, premenstrual symptoms, and health-related quality of life during the prior 6 months. At the end of the 12-week yoga exercise intervention, we administered the same questionnaire to evaluate changes in the participants’ menstrual discomfort.

### 2.3. Assessment Instruments and Definitions

#### 2.3.1. Demographics, Personal Lifestyle, Behavior, and Employment Status

Subjects’ self-reported demographics, personal lifestyle, behavior, and employment status were assessed. Employment characteristics were collected, including shift work (yes/no), worksite (office vs. clean room), and work hours per day (8 h a day or 9–14 h a day). The subjects were asked questions about their cigarette smoking status (current-smoker and never-smoker). Alcohol intake was categorized as no habit of alcohol consumption (alcohol consumption only once per week or less) and a habit of alcohol consumption (alcohol consumption more than once per week). Exercise habit included exercise that was initiated or/and maintained during the month prior to initiating the study. Subjects were asked, “Have you participated in regular exercise equivalent to a 1-h walk at least once a week (yes/no) or have you exercised at least three times per week to the extent that you breathe deeply or sweat (yes/no)”. The subjects were asked questions about their self-reported current mental status (“Does your daily life or work cause you to feel stress?” yes/no). Sleep problems were defined as difficulty falling asleep; difficulty remaining asleep (more than twice a week); the belief that one is not getting enough sleep, resulting in a disturbance of daily activities or normal social activities; and the use of medication for insomnia, focusing on symptoms during the preceding 4 weeks.

#### 2.3.2. Menstrual Status, Menstrual Pain Scores, and Self-Reported Premenstrual Symptoms

The subjects reported menstrual status information pertaining to age at menarche, menstrual regularity (cycle regularity), menstrual cycle, duration of the menstrual cycle, menstrual quantity (little, moderate, or heavy flow), self-reported perception of the effect of menstrual pain on work (no effect, little, moderate, and great), and menstrual pain (scored based on a visual analog scale (0–100 score)). Analgesic use within the last 6 months was recorded. The questionnaires included screening questions regarding 24 self-reported premenstrual symptoms within 6 months (19 physical premenstrual symptoms and five psychological premenstrual symptoms) and subjects were asked to rate the severity of premenstrual symptoms as “not at all,” “mild,” “moderate”, or “severe”. We divided subjects with premenstrual symptoms into two groups: “moderate to severe premenstrual symptoms” and “no/mild premenstrual symptoms”.

#### 2.3.3. Short-Form 36-Item Health Survey

The Short-Form 36 (SF-36) is a generic measure that assesses health concepts representing basic human values relevant to everyone’s functional status and well-being [21]. A Chinese Taiwanese version of the SF-36 was used in our study [22]. The SF-36 comprises eight aspects of health status: physical functioning, role limitations due to physical health problems, bodily pain, general health, vitality, social functioning, role limitations due to emotional problems, and mental health. Each scale are summed and rescaled to a 100-point scale, where 100 is the best possible score and 0 is the worst possible score. As such, lower scores indicate greater limitation.

### 2.4. Statistical Analysis

This study explored the effect of a 12-week yoga exercise intervention on changes in the prevalence of self-reported premenstrual symptoms, menstrual pain scores, and SF-36 scores among female employees. All analyses were performed using Statistical Analysis System software (SAS 9.3; SAS Institute, Cary, NC, USA). Descriptive characteristics of the subjects and of menstruation are expressed as percentage and mean ± SD. Association of the yoga exercise intervention with before-and-after self-reported premenstrual symptoms, menstrual status, and SF-36 scores was estimated using a McNemar’s test and paired t test. We examined the correlation between the changes in the scores of eight scales in the SF-36 and in menstrual pain scores using Pearson product-moment correlation coefficient to measure the degree of linear dependence between changes in the menstrual pain scores and changes in health-related quality of life.

## 3. Results

A total of 64 subjects completed the intervention study and the before-and-after questionnaire. Mean age of the subjects was 34.0 ± 5.5 years. Most of the subjects (56.3%) were between 31 and 40 years of age, and 62.5% had a college education level (Table 1). Among subjects, 25.0% were shift workers and 86.0% were office workers (non-clean-room workers). Only 33.4% of the subjects averaged 8 h of work per day, and mean hours of work per day was 9.6 ± 1.2 h. In addition, many of the subjects (96.9%) reported no smoking habit, 89.1% reported no alcohol drinking habit, 54.7% reported no regular exercise habit, and 44.4% subjects reported they felt stress in daily life or work. The majority (67.2%) of subjects were 11 to 15 years old at menarche (mean age of menarche was 13.6 ± 1.5 years); 17.2% reported irregular menstruation, and 75.0% reported moderate menstrual flow.

The large majority of subjects (90.6%) reported that they experienced menstrual pain during their menstrual period (Table 2). The comparison of characteristics of menstruation, exercise habit, sleep status, and SF-36 scores before the yoga exercise intervention and after 3 months revealed that subjects decreased their use of analgesics during menstruation (after: 21.9% vs. before: 35.9%, *p* = 0.0290) and the prevalence of a moderate or severe effect of menstrual pain on work was lower (after: 29.7% vs. before: 53.1%, *p* = 0.0011) after the yoga exercise intervention. Regular exercise was performed by only 45.3%, but after intervention, 71.9% subjects reported a regular exercise habit. The yoga exercise intervention was associated with the improvement of the scale of physical function (*p* = 0.0340) and bodily pain (*p* = 0.0087) of SF-36.

The 12-week yoga exercise intervention was significantly correlated with decreased prevalence of four physical symptoms, including abdominal swelling (*p* = 0.0011), breast tenderness (*p* = 0.0348), abdominal cramps (*p* = 0.0016), and cold sweats (*p* = 0.0143; (Table 3) The correlation of changes in the scores of the 8 scales of the SF-36 and menstrual pain scores at baseline and after the 3-month yoga exercise intervention is shown in (Table 4). The results demonstrated that menstrual pain mitigation after yoga exercise was correlated with improvement in six scales of the SF-36, including physical function (*r* = 0.529), bodily pain (*r* = 0.365), general health perception (*r* = 0.280), vitality/energy (*r* = 0.351), social function (*r* = 0.318), and mental health (*r* = 0.3555).

## 4. Discussion

In the present study, 82.8% participants had regular menstrual cycles. Moderate and heavy menstrual flow was reported by 85.9% of the participants and menstrual pain was reported by 90.6% of the participants. The majority of the subjects, 53.1%, reported moderate or severe effects of menstrual pain on work, with 35.9% subjects required analgesics every month during menstruation to relieve menstrual pain. None of the participants had any gynecologic disease known to induce menstrual pain, but all reported that PMS affected their well-being and quality of life. The study subjects had lower scores (i.e., greater disability) in four of the eight scales of the SF-36: bodily pain, general health perceptions, vitality/energy, and mental health. Although the decline in the quality of life caused by menstruation was not unmanageable, other factors, such as job stress, could influence the scores. These dimensions of health-related quality of life revealed that subjects were uncomfortable, felt tired or downhearted, and did not feel as healthy as they wished. Based on our results, the regular yoga exercise intervention improved the bodily pain score and physical function scores of the SF-36. In addition, four self-reported moderate or severe premenstrual symptoms (abdominal swelling, breast tenderness, abdominal cramps, and cold sweats) were significantly decreased after the 12-week yoga exercise intervention. These findings suggest that yoga exercise in the workplace effectively reduces the symptoms of PMS and can be applied to other woman-friendly workplaces to relieve PMS.

Painful menstrual periods and PMS are the most common gynecologic problems, and are the most common reasons for increased absenteeism and more workdays with 50% or less of typical productivity per month in female employees [23]. Recent studies reported an association between exercise and PMS, and indicated that a regular exercise habit might decrease some physical and psychologic premenstrual symptoms [24,25]. Pain, a common symptom of PMS, is a complex experience that affects mood and behavior, and can modify thought patterns leading to activation of different brain regions during cognitive tasks [25]. One study [25] demonstrated that women with PMS participating in a short-term yoga exercise in the luteal phase felt better and had improved attention. Another study demonstrated that the mean scores of PMS and symptoms declined after 8 weeks of aerobic exercise training in the experimental group and suggested that 8 weeks of aerobic exercise effectively reduces the symptoms of PMS and can be used as a treatment [24]. An increase in alpha wave production induced by yoga exercise is closely associated with slower abdominal breathing [25]. Yoga has positive effects on brainwave activity, and alpha brain waves are associated with states of peace, relaxation, creativity, mood elevation, and the release of serotonin; thus, the increase in alpha brain waves suggests that participants felt more relaxed after yoga exercise [26]. Other studies investigated the mitigation of PMS effects in participants that actively performed yoga postures [17,18,19]. A randomized controlled trial in India demonstrated Yoga Nidra practice was helpful in patients with hormone imbalances, such as dysmenorrhea, oligomenorrhea, menorrhagia, metrorrhagia, and hypomenorrhea [17]. Yoga poses (cobra, cat, and fish poses) reduce the severity and duration of primary dysmenorrhea, and are a safe and simple treatment for primary dysmenorrhea [18]. A yoga intervention reduces the severity of dysmenorrhea and may be effective for lowering serum homocysteine levels after an intervention period of 8 weeks [19]. These findings indicate that yoga exercise can improve menstrual pain.

As demonstrated by our data, the high prevalence of physical premenstrual symptoms (abdominal swelling, fatigue, backache, and abdominal cramps) in female employees deeply negatively affects their health-related quality of life and decreases occupational productivity. Some women, however, experience moderate or severe symptoms that are emotionally disabling, particularly in the area of personal relationships and social activities [1]. A Swiss population-based health survey revealed that 57% women report having at least a mild degree of “premenstrual anger/irritability” or “premenstrual tearfulness/mood swings”; the median duration of physical and emotional symptoms is 3 days, and relationships with co-workers and/or family are most affected [6]. In the present study, female workers reported experiencing irritability, emotional lability, and depressed mood. The psychologic symptoms can interfere with interpersonal relationships, social interactions, and emotional well-being in the workplace. Our findings (Table 4) indicated that when menstrual pain was relieved, most dimensions of the SF-36 were greatly enhanced, including physical function, bodily pain, general health perception, vitality/energy, social function, and mental health. A previous study of the use of a DVD to provide a simple home-based yoga program also demonstrated improvements of menstrual pain and overall health status [20]. A systematic review of five studies that examined three psychologic mechanisms (positive effect, mindfulness, and self-compassion) and four biologic mechanisms (posterior hypothalamus, interleukin-6, C-reactive protein. and cortisol), revealed that positive affect, self-compassion, and inhibition of the posterior hypothalamus and salivary cortisol mediated the effects of yoga on stress [27]. One report exploring the effects of yoga on persistent pain indicated that yoga could produce psychologic changes, such as increased awareness of mental and physical states, which may help patients to better understand their pain. Therefore, yoga practice might lead to increased pain acceptance—the willingness to experience pain and acknowledge negative thoughts and emotions. It is also possible that yoga improves self-efficacy for pain control [28]. Based on these positive findings, workplaces and employers can help female workers to understand the benefits of yoga exercise, which may decrease premenstrual distress and improve the health of female workers, especially those who suffer from moderate or severe PMS.

Our study has some limitations. First, the study was a simple experimental design comparing before and after a single intervention; therefore, the results were not obtained using control groups or a random allocation design. Further, no temporal relations could be assessed, and only associations, not causation, were evaluated. Second, our assessment of independent factors using a dichotomized classification may be overly simplistic. Third, questions of demographics, lifestyle, menstrual characteristics, and perceived self-reported premenstrual symptoms within 6 months in the questionnaire were developed for this study; therefore, the fact that standard instruments were not applied in the present study could have led to a misclassification of information, and might have also affected the validity and reliability of questionnaire. Fourth, a selection bias due to a healthy-subjects effect was inevitable and the low participation rate may have led to biased results of the study and reflect an imprecise estimation due to the relatively small sample size. The small sample size reduces the strength of the established associations. The drop-out rate from the study was quite high (198 participants began the study, and only 64 completed it) and one reason for this may be the high workload. Among employees in the study sample, only 34.4% of the participants averaged 8 h of work per day and the average number of work hours per day of all participants was 9.55 ± 1.23. Long working hours or a heavy workload may influence participants’ intention to complete the 12-week intervention. The yoga intervention was scheduled after work and not during the work day. Finally, we did not explore whether participants had prior yoga exercise experience or whether they performed more yoga exercise, which could affect the results.

Additional studies should be performed with control groups or a random allocation design to implement and adapt standard instruments regarding premenstrual symptoms and menstrual status information. Use of standard instruments can ensure higher validity and reliability of the questionnaire and allow for comparison of the results with other studies. Furthermore, potentially confounding factors, such as prior yoga exercise experience and whether participants practiced yoga exercise at home during the study period should be investigated. Finally, future studies should compare the impact of different yoga protocols.

## 5. Conclusions

In conclusion, in the present study, female employees participating in a short-term yoga exercise intervention reported fewer physical premenstrual symptoms associated with a lower risk of menstrual pain. Workplaces and employers can help female employees to understand the benefits of regular exercise, such as yoga, which may decrease premenstrual distress and improve the health of female employees.

## Figures and Tables

**Table 1:** Descriptive characteristics of 64 study participants.

Variable (Mean ± SD)	Category	Number	%
Age, years, (34.0 ± 5.5)	21–30	18	28.1
	31–40	36	56.3
	41+	10	15.6
Education	High school	4	6.3
	College	40	62.5
	Graduate studies	20	31.2
Shift work	No	48	75.0
	Yes	16	25.0
Worksite	Clean room	9	14.0
	Office	55	86.0
Work hours per day, (9.55 ± 1.23)	<9 h	22	34.4
	≥9 h	42	65.6
Smoking	Yes	2	3.1
	No	62	96.9
Alcohol	Yes	7	10.9
	No	57	89.1
Exercise habit	Yes	29	45.3
	No	35	54.7
Daily life or work feel strenuous	Yes	28	44.4
	No	36	55.6
Age at menarche, (13.6 ± 1.53)	9–10 years old	4	6.3
	11–15 years old	43	67.2
	>15 years old	17	26.6
Menstruation regularity	Regular	53	82.8
	Irregular	11	17.2
Menstruation amount	Little	9	14.1
	Moderate	48	75.0
	Heavy	7	10.9

**Table 2:** Comparison of characteristics of menstruation, exercise habit, sleep status, and SF-36 scores at baseline and after 3 months yoga exercise intervention.

Variables	Participants		*p*-Value (McNemar’s Test)
	Baseline (%)	After 3 Months (%)	
Characteristics of menstruation			
Menstruation regularity (Irregular)	17.2	17.2	1.0000
Menstruation amount (Heavy)	10.9	7.8	0.4142
Menstrual pain (Yes)	90.6	89.1	0.7630
Take analgesics medicine every month during menstruation (Yes)	35.9	21.9	0.0290
Impact of menstrual pain on work (Self-reported moderate or severe)	53.1	29.7	0.0011
Menstrual pain scores (mean ± SD)	56.82 ± 24.0	44.8 ± 25.7	0.0004 (Paired *t* test)
Exercise habit (Yes)	45.3	71.9	0.0004
Sleep status (bad)	20.3	12.5	0.1655
SF-36			
Physical function	84.6 ± 25.0	91.3 ± 12.1	0.0340 (Paired *t* test)
Role limitations: physical	83.2 ± 31.8	80.8 ± 35.5	0.6763 (Paired *t* test)
Bodily pain	61.3 ± 20.5	68.9 ± 18.7	0.0087 (Paired *t* test)
General health perceptions	55.9 ± 14.7	55.5 ± 16.9	0.7995 (Paired *t* test)
Vitality/energy	54.6 ± 14.5	53.6 ± 13.8	0.6557 (Paired *t* test)
Social function	76.5 ± 16.7	77.1 ± 16.4	0.8795 (Paired *t* test)
Role limitations: emotional	76.5 ± 38.3	72.9 ± 37.9	0.5480 (Paired *t* test)
Mental health	61.2 ± 12.5	62.3 ± 12.2	0.5452 (Paired *t* test)

**Table 3:** Comparison of self-reported moderate or severe frequency of premenstrual symptoms among study participants and exercise habit by moderate or severe symptoms.

Variables	Self-Reported Moderate or Severe Frequency of Premenstrual Symptoms		*p*-Value (McNemar’s Test)
	Baseline	After 3 Months	
*Physical symptoms*			
Muscle stiffness	7.81	15.6	0.1655
Faintness	0	1.56	--
Abdominal swelling	50.0	23.4	0.0011 *
Dizziness, fuzzy version	12.5	15.6	0.5271
Breast tenderness	20.3	9.4	0.0348 *
Easy to fatigue	48.4	39.0	0.1797
Abdominal cramps	42.9	21.9	0.0016 *
Leg swelling	19.0	10.9	0.1317
Backache	48.4	37.5	0.1266
Somatic discomforts	25.0	17.2	0.1967
Headache	28.1	23.4	0.3657
Palpitation	3.1	1.6	0.3333
Skin allergies, itch	14.1	10.9	0.5271
Cold sweats	12.5	3.1	0.0143 *
Nausea, vomiting	6.3	6.2	0.9999
Hot flashes	3.1	3.1	--
Diarrhea	14.1	10.9	0.5271
Constipation	12.5	4.7	0.0956
Weight gain	14.1	10.9	0.5271
*Psychological symptoms*			
Irritability	25.0	15.6	0.1573
Feeling depressed	14.1	15.6	0.7815
Crying	7.8	6.3	0.7055
Tension	10.9	10.9	1.0000
Emotional lability	20.3	10.9	0.1573

*: *p* < 0.05.

**Table 4:** Correlation of score changes in the 8 scales of the SF-36 and menstrual pain scores between baseline and after 3 months yoga exercise intervention.

Variable	Positive Score Changes in the 8 Scales of the SF-36 between Baseline and after 3 Months Yoga Exercise Intervention (after 3 Months Scores—Baseline Scores)							
	Physical Function	Role Limitations: Physical	Bodily Pain	General Health Perceptions	Vitality/Energy	Social Function	Role Limitations: Emotional	Mental Health
Positive changes in menstrual pain scores (scores at baseline—scores after 3 months)	0.529 (*p* < 0.0001)	0.237 (*p* > 0.05)	0.365 (*p* = 0.0032)	0.280 (*p* = 0.0257)	0.351 (*p* = 0.0051)	0.318 (*p* = 0.0124)	0.0213 (*p* > 0.05)	0.3555 (*p* = 0.004)
